# Editorial: Metal-free room-temperature phosphorescence

**DOI:** 10.3389/fchem.2022.1025674

**Published:** 2022-09-23

**Authors:** Shuzo Hirata, Sebastian Reineke, Abhijit Patra, Wang Zhang Yuan

**Affiliations:** ^1^ The University of Electro-Communications, Tokyo, Japan; ^2^ Dresden Integrated Center for Applied Physics and Photonic Materials (IAPP), Technische Universität Dresden, Dresden, Germany; ^3^ Indian Institute of Science Education and Research Bhopal, Bhopal, India; ^4^ Shanghai Jiao Tong University, Shanghai, China

**Keywords:** room-temperature phosphorescence, metal-free RTP, persistent RTP, delayed emission, triplet state, nonradiative deactivation, oxygen quenching, intersystem crossing

Room-temperature phosphorescence (RTP) from metal-free materials has recently attracted the interest of the scientific community in a variety of imaging fields owing to the fact that it provides high-resolution information independent of the autofluorescence and dual-colored emitting characteristics, which are advantages that fluorescent materials and phosphorescent heavy-metal complexes do not possess. State-of-the-art advances in security, sensors, and bioimaging have been proposed using the aforementioned advantages. This Research Topic includes ten articles on the latest advances in this research area.

Suppression of triplet deactivation, including oxygen quenching, is an important factor to enhance the RTP properties. An article by (Yang et al.) reports oxygen sensing using thianthrene derivatives doped into amorphous polymer matrices. The thianthrene chromophores show dual emission of fluorescence and RTP in the polymers because folding-induced spin–orbit coupling of the chromophores greatly enhances the RTP characteristics. Ratiometric imaging owing to the dual fluorescence and the enhanced RTP allows easy quantitative detection of oxygen under a variety of conditions. Furthermore, the relationship between the RTP intensity and oxygen concentration provides quantitative information about the oxygen permeability in solid materials.

As different triplet-quenching characteristics independent of oxygen, a study by (Thomas et al.) investigates the change of the triplet nonradiative processes of RTP chromophores doped in a polymer host under inert conditions depending on different preparation techniques of the films. Residual solvents and the role of guest chromophores in enhancing the intermolecular interactions of the polymer matrices are proposed as reasons for the different nonradiative deactivation from the triplet state. For the interaction between the guest chromophore and polymer host, the tendency of smaller triplet deactivation using polymers with lower molecular weights is also introduced.

A study by (Kusama and Hirata) investigates the nonradiative process from the triplet state of guest chromophores under the oxygen-free condition from the viewpoint of the intramolecular nonradiative transition of the guest and the endothermic intermolecular triplet quenching between the guest and molecular host. Experiments using large different persistent RTP yield depending on thermo-reversible amorphous/crystal changes of the host–guest solid reveal that the different diffusion constants of the molecular host are largely related to the magnitude of the endothermic intermolecular triplet quenching.

As another article introducing suppressed triplet deactivation, Zhou et al. explains the details of the RTP characteristics of cotton fibers. The clustering of the non-aromatic units in the cotton fibers is explained as the generation source of the RTP, and the RTP characteristics are observed in the central part of the fibers. An increase of the concentration of sodium ions is explained to increase the rigidity, which reduces nonradiative deactivation from the triplet state.

The topic of triplet deactivation is also related to RTP from carbon nanodots. Patir and Gogoi show the matrix-free RTP characteristics of chlorine-doped carbon nitride dots (CNDs) in air. The authors explain that the C–Cl or C–NH_2_ groups on the surface of the CNDs provide rigidity via hydrogen bonding, reducing the nonradiative transition from the triplet state. This allows persistent RTP even when the CNDs are not surrounded by a solid matrix.

Research regarding the triplet generation yield is also crucial for enhancing the RTP characteristics. Vasilev et al. explains efficient triplet generation by photoexcitation of synthesized porphyrin derivatives using the photon up-conversion properties. Although the triplet-generation capability is often small for heavy-atom-free chromophores with a small energy gap, an adequate triplet generation yield is obtained for the synthesized heavy-atom-free porphyrins.

As other report focusing on the contribution of triplet generation to the RTP characteristics, Cao et al. introduces benzophenone (BPO) doped with thianthrene (TTR). Analysis using different concentrations of TTR indicates that the triplet–triplet energy transfer from the BPO host to the TTR guest after excitation of BPO contributes to enhance the RTP yield. A TTR/BPO material with an optimized TTR concentration shows RTP with 46% quantum efficiency and a lifetime of 9.17 ms from isolated TTR under ambient conditions.

Research regarding the relationship between the RTP characteristics and the conjugated backbone may be crucial for providing additional insight into enhancing the RTP characteristics, Takewaki et al. reports mechano/thermoresponsive RTP chromism from solids of thienyl diketone derivatives. Although thienyl diketone derivatives form the skew conformer generate green RTP in the crystalline state of the solid, an amorphous state containing some of the thienyl diketone derivatives shows yellow RTP characteristics after grinding. After heating of the amorphous state, the crystalline state with green RTP characteristics is recovered.

9,10-Diphenylfluorescence derivatives substituted with bromine as guest chromophores showing delayed emission characteristics are reported by (Zhang et al.). The derivatives show different delayed emission spectra in the crystalline state, doped state in the amorphous polymer, and dissolving state in degassed organic solvents. The delayed emission has multi-exponential decay characteristics, and the magnitude of the average time of decay is from 1 µs to 100 ms.

In addition to the RTP characteristics, utilization of stabilized triplet excitons is also crucial for expanding applications. Yang et al. prepared a co-crystal composed of 9,10-di (4-carboxyphenyl)anthracene (DCPA) and acridine (AD) [(DCPA) (AD)_2_]. [(DCPA) (AD)_2_] shows long-lived triplet excitons as the RTP characteristics with a lifetime of 325 μs. The authors propose that the long-lived triplet state, as well as separation of the molecule orbitals, between DCPA and AD of the crystals contributes to a greater change of the long-distance exciton transfer and good electron–hole separation ability, resulting in excellent photoelectric response performance.

Although metal-free RTP materials and their applications have been reported recently, the articles in this themed issue provide proposals and novel insight for previously unclear points regarding the photophysical processes related to the triplet state from the viewpoint of RTP. The editors believe that this Research Topic of articles will inspire readers to consider the idea of improvement of a variety of opto-electronics and photonics applications, as well as enhancement of the RTP characteristics.

